# Conductive extracellular matrix derived/chitosan methacrylate/ graphene oxide-pegylated hybrid hydrogel for cell expansion

**DOI:** 10.3389/fbioe.2024.1398052

**Published:** 2024-06-17

**Authors:** Valentina Jaramillo, Daniel Felipe Arévalo, Martin González-Hernández, María T. Cortés, Ana María Perdomo-Arciniegas, Juan C. Cruz, Carolina Muñoz-Camargo

**Affiliations:** ^1^ Grupo de investigación en Nanobiomateriales, Ingeniería Celular y Bioimpresión (GINIB), Departamento de Ingeniería Biomédica, Universidad de los Andes, Bogotá, Colombia; ^2^ Department of Chemistry, Universidad de los Andes, Bogotá, Colombia; ^3^ Cord Blood Bank (CBB) Research Group, Instituto Distrital de Ciencia, Biotecnología e Innovación en Salud (IDCBIS), Bogotá, Colombia

**Keywords:** electrostimulation, electroconductive nanomaterials, hydrogels, 3D cell culture, cell proliferation

## Abstract

Electrical stimulation has emerged as a cornerstone technique in the rapidly evolving field of biomedical engineering, particularly within the realms of tissue engineering and regenerative medicine. It facilitates cell growth, proliferation, and differentiation, thereby advancing the development of accurate tissue models and enhancing drug-testing methodologies. Conductive hydrogels, which enable the conduction of microcurrents in 3D *in vitro* cultures, are central to this advancement. The integration of high-electroconductive nanomaterials, such as graphene oxide (GO), into hydrogels has revolutionized their mechanical and conductivity properties. Here, we introduce a novel electrostimulation assay utilizing a hybrid hydrogel composed of methacryloyl-modified small intestine submucosa (SIS) dECM (SISMA), chitosan methacrylate (ChiMA), and GO-polyethylene glycol (GO-PEG) in a 3D *in vitro* culture within a hypoxic environment of umbilical cord blood cells (UCBCs). Results not only demonstrate significant cell proliferation within 3D constructs exposed to microcurrents and early growth factors but also highlight the hybrid hydrogel’s physiochemical prowess through comprehensive rheological, morphological, and conductivity analyses. Further experiments will focus on identifying the regulatory pathways of cells subjected to electrical stimulation.

## 1 Introduction

Hydrogels have played an important role in advancing the fields of tissue engineering and regenerative medicine, owing to their pivotal role in developing accurate tissue models and facilitating drug testing (Cascone and Lamberti, 2020). Their unique structure, hydrophilic properties, and versatile composition render them an ideal platform for designing and manufacturing cell-friendly and multifunctional microenvironments (Ahadian et al., 2016; Serna et al., 2021). Hydrogels composed of naturally derived polymers such as fibrin, hyaluronic acid, and collagen offer biocompatibility and biochemical similarities to natural tissue microenvironments. Decellularized extracellular matrices (dECM) are particularly noteworthy for their ability to support crucial cellular processes such as proliferation, differentiation, migration, and spatial organization (Zhang et al., 2005; Sánchez-Palencia et al., 2014; Rueda-Gensini et al., 2021), while retaining the biochemical properties of their original tissue microenvironments (Tsui et al., 2021).

Despite their widespread use, the mechanical fragility of these naturally derived polymers poses significant challenges. These materials often require stabilization through chemical modifications to form robust structures capable of enduring physiological conditions. Methacryloyl-modified SIS dECM hydrogels (SISMA), derived from the porcine small intestine submucosa (SIS), exemplify such an innovation (Sánchez-Palencia et al., 2014; Rueda-Gensini et al., 2021). These hydrogels (dECM-SISMA) comprise essential proteins commonly found in tissue ECMs, including collagen I, III, and XI, fibronectin, laminin, proteoglycans, and elastin. On the other hand, chitosan methacrylate (ChiMA), a modified natural polymer derived from chitin, possesses strong adhesiveness and biocompatibility due to its positively charged surface. This characteristic promotes cell attachment and differentiation by mimicking constituents of the natural ECM (Wise et al., 2016; Re et al., 2019; Céspedes-Valenzuela et al., 2021).

The advent of nanocomposite hydrogels has introduced new possibilities for enhancing the mechanical and conductivity properties of dECMs. By incorporating high-electroconductive nanomaterials, such as carbon nanotubes, graphene derivatives, and gold, into hydrogels, researchers have made significant strides in understanding cell growth and maturation processes (Tsui et al., 2021; Dutta et al., 2023). Graphene, a 2D material comprised of coplanar sp^2^-hybridized carbon atoms, has been harnessed in the biomedical field for its electroconductive properties, mechanical properties, and ability to deliver therapeutic agents (Dutta et al., 2023). Graphene oxide (GO), an oxidized variant of graphene, features polar functional groups, including carboxyl, hydroxyl, carbonyl, and epoxy groups. These functional groups offer improved colloidal stability in water and covalent functionalization opportunities. When combined with polyethylene glycol (PEG), GO’s physiological stability is enhanced, facilitating cellular uptake, improving loading, and releasing characteristics into aqueous matrices and reducing cytotoxicity, thus paving the way for the development of conductive hydrogels capable of electrical stimulation (Raslan et al., 2020; Khramtsov et al., 2021).

3D-tissue modeling is an attractive strategy for hematopoietic stem and progenitor cell expansion, physiological studies, drug testing, disease modeling, and advanced therapy design (Xiao et al., 2022). The hematopoietic niche has a substantial cellular diversity (hematopoietic and non-hematopoietic) within a relatively hypoxic microenvironment, providing conditions for aerobic glycolysis used mainly by long-term stem and progenitor cells to proliferate.

This manuscript presents a novel approach to creating a hybrid, electro-stimulable hydrogel comprising methacryloyl-modified SIS dECM (SISMA), chitosan methacrylate (ChiMA), and GO-PEG. This innovative hydrogel is designed for hematopoietic stem and progenitor cell expansion within 3D *in vitro* hypoxic cultures, leveraging electrical stimulation to promote cell growth and proliferation through mechanisms such as voltage-gated ion channel activation and MAPK pathway activation, among others (Ahadian et al., 2016; Katoh, 2023). This work not only introduces a robust method for synthesizing this hybrid hydrogel but also highlights its potential in advancing the field of tissue engineering and regenerative medicine.

## 2 Experimental section

### 2.1 Materials

Sulfuric acid 98% (v/v) (H_2_SO_4_), phosphoric acid 85% (v/v) (H_3_PO_4_), potassium permanganate (KMnO_4_), hydrogen peroxide 30% (v/v) (H_2_O_2_), hydrochloric acid (v/v) HCl, glacial acetic acid, sodium hypochlorite (NaClO), Tris-HCl, ethanol 96% (v/v), and sodium hydroxide (NaOH) were purchased from PanReac AppliChem (Chicago, IL, United States). High-density chitosan, phosphate-buffered saline (PBS), porcine pepsin, methacrylic acid (MA), N-[3-(dimethylamine)-propyl]-N′-ethyl carbodiimide hydrochloride (EDC), N-hydroxysulfosuccinimide (NHS), dimethylformamide (DMF), chloroacetic acid (Cl-CH_2_-COOH), polyethylene glycol (PEG) 4-Arm-NH2, and LAP were purchased from Sigma-Aldrich (St. Louis, MO, USA). RPMI 1640 medium was purchased from Gibco (Amarillo, TX, United States). Flt3 ligand, thrombopoietin, and stem cell factor (SCF) were purchased from R&D Systems (Minneapolis, MN, United States). The mix of penicillin and streptomycin was purchased from BioWest. alamarBlue™ reagent, calcein-AM, and propidium iodide were purchased from Invitrogen (Waltham, MA, United States). HES 6% solution was purchased from Grifols (Laboratorios Grifols SA, Barcelona, Spain).

### 2.2 Graphene oxide (GO) synthesis and PEGylation chemical modification

#### 2.2.1 Graphene oxide (GO) synthesis

GO was synthesized using the Tour method, which involves the coupled exfoliation and oxidation of graphite (Marcano et al., 2010). A 9:10 mixture of H_2_SO_4_ and phosphoric acid (H_3_PO_4)_ was slowly added to a flask containing 0.75 g graphite flasks and 4.5 g KMnO_4_ in an ice bath. Then, the reaction was left at 50°C for 12 h under magnetic stirring. Subsequently, ice-cold Type I H_2_O and 3 mL H_2_O_2_ were added to the reaction mixture. The resulting GO was then filtered using polyester fiber, centrifuged at 4000 rpm for 4 h, and washed twice with a solution composed of 50 mL HCl, 50 mL ethanol, and 50 mL Type I H_2_O. The final pellet was resuspended in Type I H_2_O and subjected to a 24-h lyophilization process. Finally, the correct synthesis was confirmed through Fourier-transform infrared spectroscopy (FTIR) in the spectral range of 4,000–400 cm^−1^. The synthesis was also confirmed by collecting the Raman spectra to assess the excitation-emission intensity difference within a range of 0 and 300 Raman shift (cm^−1^), with point-wise laser excitation at 532 nm, and a thermogravimetric analysis (TGA) thermogram using a temperature ramp from 25°C to 800°C at a heating rate of 10°C/min under a nitrogen atmosphere.

#### 2.2.2 PEGylated graphene oxide (GO-PEG) chemical modification

Post-synthesis, GO underwent PEGylation through carbodiimide-mediated coupling, following the protocol described by Khramtsov et al. (2021). First, a solution of the GO was prepared Type I H_2_O at a concentration of 2 mg/mL. Subsequent to carboxylate GO, Cl-CH2-COOH (to achieve a final concentration of 18.5 mM) was added during sonication for 60 min in an ultrasonic bath at an amplitude of 38% and a frequency of 40 Hz at 4°C. The carboxylated GO nanoparticles were then subjected to centrifugation at 4000 rpm and washed with Type I H_2_O until a neutral pH was achieved. Next, a mixture of EDC (4 mM), NHS (10 mM), and PEG 4 Arm (2 mg/mL) was added to the suspension under sonication. The resulting suspension was left under reaction for 24 h at RT, followed by purification by dialysis, three washes with ethanol by centrifugation, and final lyophilization. Finally, the correct synthesis was confirmed through FTIR in the spectral range of 4,000–400 cm^−1^. The synthesis was also confirmed by collecting the Raman spectra to assess the excitation-emission intensity difference within a range of 0 and 300 Raman shift (cm^−1^), with a point-wise laser excitation at 532 nm, and a thermogravimetric analysis (TGA) thermogram using a temperature ramp from 25°C to 800°C at a heating rate of 10°C/min under a nitrogen atmosphere.

### 2.3 Porcine small intestine submucosa (SIS) isolation, decellularization, and methacryloyl modification (SISMA)

Porcine small intestines were acquired from a local butcher shop, ensuring the freshness and quality of the specimens. Subsequently, the SIS was extracted and subjected to a decellularization process, following the procedure initially outlined by Sánchez-Palencia et al. (2014) and Rueda-Gensini et al. (2021) and subsequently modified by us.

#### 2.3.1 Porcine small intestine submucosa (SIS) isolation and decellularization

First, healthy segments of approximately 10 cm in length were selected from the jejunum part of the small intestine. The segments were washed with tap water to remove debris and residual contents. Then, the submucosa layer was isolated mechanically by carefully removing the tunica mucosa, muscularis, and serosa layers, ensuring the integrity of the SIS. Subsequently, the isolated tissue was decellularized by immersion in a solution comprising 8.5 mL H_2_O_2_, 50 mL NaClO, and 442 mL autoclaved Type II H_2_O under constant agitation for 20 min. Following this, the isolated tissue was rinsed three times. The submucosa tissue was autoclaved with 500 mL Type II H_2_O and 500 mL PBS 1X to remove any residual decellularization agents and cellular remnants. Finally, the submucosa samples were subjected to lyophilization for 48 h and pulverized in a cryogenic mill, yielding a fine powder.

#### 2.3.2 Methacryloyl modification of SIS (SISMA)

The SIS was chemically modified by attaching methacryloyl groups (SISMA) to the glycine residues in the collagen structure, following the procedure outlined by Rueda-Gensini et al. (2021). First, the pulverized SIS was dissolved at a concentration of 4 mg/mL in a solution comprising 1 mg/mL of porcine pepsin in 0.5 M acetic acid. This mixture was stirred magnetically for 48 h at room temperature to achieve full solubilization of SIS. Then, a modification solution of MA (in a 1:20 M excess relative to glycine residues in SIS), EDC (1:1 M ratio with MA), and NHS (1:1 M ratio with MA) was prepared in 3 mL in DMF and heated to 40°C for 15 min under constant magnetic stirring to activate the methacryloyl groups for efficient binding. Subsequently, the activated modification solution was added to the solubilized SIS, maintaining the reaction under constant magnetic stirring at 4°C for 24 h. The resulting modified SIS was then dialyzed against 0.25 M acetic acid to remove unreacted agents and byproducts, ensuring the purity of SISMA. This was followed by a 48-h lyophilization process and sterilization using ethylene oxide. Finally, the successful conjugation of methacryloyl groups to SIS was confirmed by conducting FTIR within the spectral range of 4,000–400 cm^−1^.

### 2.4 Methacryloyl biochemical modification of chitosan (ChiMA)

Chitosan was chemically modified involving the attachment of methacryloyl groups (ChiMA) to the free amines of the chitosan monomers following the protocol by Shen et al. (2020) and Céspedes-Valenzuela et al. (2021). First, high molecular weight chitosan was solubilized to achieve a 3.5 mg/mL concentration in 0.17 M acetic acid by stirring this solution continuously at RT for 10 min. Subsequently, a reactive solution of MA (1:1 M ratio), EDC (1:2 M ratio), and NHS (1:4 M ratio) with respect to the free amines of chitosan was prepared in 3 mL of DMF. The reactive solution was heated to 40°C for 15 min with constant magnetic stirring to activate the methacryloyl groups for efficient bonding, and the activated reactive solution was added to the solubilized chitosan, maintaining the mixture under magnetic stirring at 4°C for 24 h. The resulting solution was subsequently dialyzed against 0.17 M acetic acid, followed by a 48-h lyophilization process and sterilization process using ethylene oxide. Finally, the successful conjugation of methacryloyl groups to chitosan was confirmed by conducting FTIR within the spectral range of 4,000–400 cm^−1^.

### 2.5 Preparation of the hybrid SISMA-ChiMA-GO-PEG hydrogel

Initially, the RPMI 1640 medium was prepared with early-acting cytokines Flt3-L, SCF, and TPO at 50 ng/mL and penicillin/streptomycin 0.2% (v/v). A working solution with LAP 0.3% (v/v) and Tris-HCl 0.1 M at a pH of 8.5 was mixed in the previously prepared RPMI 1640 medium. Then, the working solution was filtered through a 0.22 µm filter. Subsequently, the GO-PEG nanomaterial at a concentration of 0.25 mg/mL was dispersed into the working solution and allowed to sit for 12 h at 4°C. Following this, the sterile SISMA and ChiMA were resuspended separately in a 0.02 M sterile acetic acid solution at a volume ratio of 1:4 for SISMA at 4% (w/v) and ChiMA at 1% (w/v). Then, the SISMA and ChiMA suspensions were homogeneously mixed to form the pre-gel base of the hydrogel. Finally, the working solution was added to the pre-gel at 4°C in a 1:1 volume ratio to form a hybrid hydrogel comprising SISMA 2% (w/v), ChiMA 0.5% (w/v), and GO-PEG 0.125 mg/mL.

### 2.6 Characterization of SISMA-ChiMA-GO hydrogels

#### 2.6.1 Rheological characterization

To evaluate the rheological response of the hydrogels and their response to photocrosslinking, a Discovery Series Hybrid Rheometer-1 (TA Instruments, New Castle, DE, United States) was used with a parallel plate geometry, a 20 mm gap, and approximately 1 mL of the sample. For flow sweep experiments, the hydrogel’s behavior was evaluated over a shear rate range of 0.01–200 1/s at a constant strain of 1% at RT. For time sweep experiments, the hydrogel’s rheological response to photocrosslinking was examined before and after exposing the hydrogel to blue light (5 min at 62 mW/cm^2^), using oscillatory mode at a constant strain of 1% and a frequency of 10 rad/s, at RT. Finally, the viscosity ƞ) *versus* shear rate 𝛾) were fitted to the power-law model (Eq. 1) to determine the hydrogel’s shear-thinning properties, indicative of its suitability for applications requiring injectability or precise deposition:

$$ƞ=K\gamma^{ƞ-1}$$ (1)

#### 2.6.2 Texture profile analysis

The texture profile analysis for each hydrogel was conducted using a TA.HDplusC texture analyzer from Stable Micro Systems (Godalming, UK) to evaluate the texture profile of each hydrogel sample, with assessments conducted both pre- and post-irradiation, adhering to the methodology outlined by Céspedes-Valenzuela et al., 2021. Initially, the hydrogel samples were shaped into cylindrical constructs, ensuring each had a diameter of 20 mm and a height of 25 mm. Then, the compression force was measured using a cylindrical probe (10 mm in diameter) at a constant speed of 1.0 mm/s, penetrating the hydrogel to a depth of 15 mm. Finally, the hardness, adhesiveness, compressibility, and cohesiveness were obtained from the texture-profile graph (Supplementary Figure S1) following the equations. F1 is the highest peak force measured during the first compression (Eq. 2), area 2 is the area under the curve for the first negative peak (Eq. 3), area 1 is the area underneath the first compression (Eqs 4, 5), and area 3 is the area underneath the second compression (Eq. 4):

Hardness=F1 (2)

Adhesivness=Area 3 (3)

Compressibility=Area 2/Area 1 (4)

Cohesiveness=Area 1 (5)

#### 2.6.3 Morphological analysis by scanning electron microscopy (SEM)

The hydrogel microstructure of SISMA 2% w/v–ChiMA 0.5% w/v with GO-PEG 0.125 mg/mL hydrogel was evaluated through SEM imaging (JSM 6490-LV, JEOL, Tokyo, Japan). First, the hybrid hydrogel composed of SISMA (2% w/v), ChiMA (0.5% w/v), and GO-PEG (0.125 mg/mL) was prepared and subjected to photocrosslinking using blue-light irradiation (5 min at 62 mW/cm^2^ intensity) to solidify its structure. Then, the photocrosslinked hydrogel samples were lyophilized (freeze-dried). Finally, the freeze-dried samples were examined using an SEM under vacuum conditions, operating at 200× magnification and an accelerating voltage of 20 kV.

#### 2.6.4 Electrochemical analysis

For electrochemical measurements of the SISMA 2% w/v–ChiMA 0.5% w/v and SISMA 2% w/v–ChiMA 0.5% w/v with GO-PEG 0.125 mg/mL hydrogels, cyclic voltammetry (CV) and impedance spectroscopy (EIS) was conducted using a three-electrode set up and a reference electrode (Ag/AgCl), glassy carbon as a working electrode, and a counter electrode (Pt wire). Initially, the glassy carbon electrode was polished with alumina paste and then sonicated to ensure a clean and reactive surface. Carefully, 25 μg of the hydrogel was deposited onto the working electrode, and the hydrogel was allowed to dry completely before the CV and EIS measurements. Then, CV measurements were conducted on the hydrogel samples at a potential window of −0.6 V–0.9 V and a scan rate of 100 mVs^−1^. Finally, the electrochemical impedance was measured at 0.9 V and a frequency from 0.1 to 10^5^ Hz. The specific capacitance was calculated for every variation of the CV curves following Equation 6:

$$C=\frac{Q}{2\;V_{m}}$$ (6)

### 2.7 Proof-of-concept: Bioink preparation and electrostimulation assay

Umbilical cord blood (UCB) units were collected at different local hospitals (Meissen, Kennedy, and Suba) and the Cafam Clinic in Bogotá Capital District (Colombia) from consenting healthy mothers. The study received ethical approval from the Ethical Committee of the Secretaría Distrital de Salud (District Secretary of Health) and Universidad de los Andes (Minute No. 1421, 2021).

#### 2.7.1 Mononuclear cells from umbilical cord blood (UCBMCs) isolation

Initially, mononuclear cells from umbilical cord blood (UCBMCs) were isolated by using the Ficoll-Paque (LymphoprepTM, Stemcell Technologies, Canada) density gradient procedure. UCBMCs were homogenized with 90% of sterile autologous neonatal plasma and 10% of CryoSure-DEX40 (Wak-Chemie Medical GMbH) at a concentration of 4 × 10^7^ cells/mL, maintaining the temperature at 4°C during the CryoSure-DEX40 addition. Then, UCBMCs were cryopreserved using a freezing curve ranging from 4°C to 0°C (4°C/min), to −10°C (1°C/min), to −50°C (20°C/min), to −18°C (15°C/min), to −40°C (1°C/min) to −60 °C (2°C/min) and to −80°C (3°C/min) in a CryoMed Freezer (Thermo Scientific, Waltham, Massachusetts, United States). After reaching −80 °C, the cryovials were stored in a vapor phase of liquid nitrogen (−190°C) tank, and their temperature was constantly monitored.

#### 2.7.2 Mononuclear cells from umbilical cord blood (UCBMCs) thawing

First, the thawing medium was prepared with sterile 69% (v/v) PBS 1X, 21% (v/v) human albumin, and 10% (v/v) HES solution 6%. Subsequently, the vials were thawed by immersion in a 37°C water bath for 5 min within a sterile zip-lock bag. A 1.5 mL aliquot of the vial of UCBMC was homogenized in a 15 mL tube with 1.5 mL of thawing medium. Then, it was incubated for 10 min at 37°C. Finally, 9 mL of thawing medium was added to the tube and incubated for 30 min at RT for cell stabilization.

#### 2.7.3 Biofabrication of the UCBMC cell-laden SISMA-ChiMA-GO-PEG constructs

Initially, the SISMA-ChiMA-GO-PEG hydrogel was prepared under sterile conditions. Then, the UCBMC cells were incorporated into RPMI 1640 medium containing early growth factors at a concentration of 50 ng/mL (Flt3-L, SCF, TPO), along with 0.2% (v/v) P/S, at a volume ratio of 1:10 with the hydrogel formulation, achieving a final cell density of 4 × 10^6^ cells/mL. Then, the bioink was extruded manually to form constructs with a final volume of 120 μL in a 24-wells culture plate. The constructs were crosslinked using 405 nm blue-light irradiation at a power of 62 mW/cm^2^ for 1 min. Subsequently, 600 μL of cell medium RMPI 1640 containing early growth factors at a concentration of 50 ng/mL (Flt3-L, SCF, TPO) was added, along with 0.2% (v/v) P/S. Finally, the constructs were incubated for up to 14 days under two different conditions: 5% CO_2_ and 5% O_2_ (hypoxic conditions) and 21% O_2_ (normoxic conditions). Each treatment was carried out in duplicate.

#### 2.7.4 Electrostimulation assay

**FIGURE 1:**
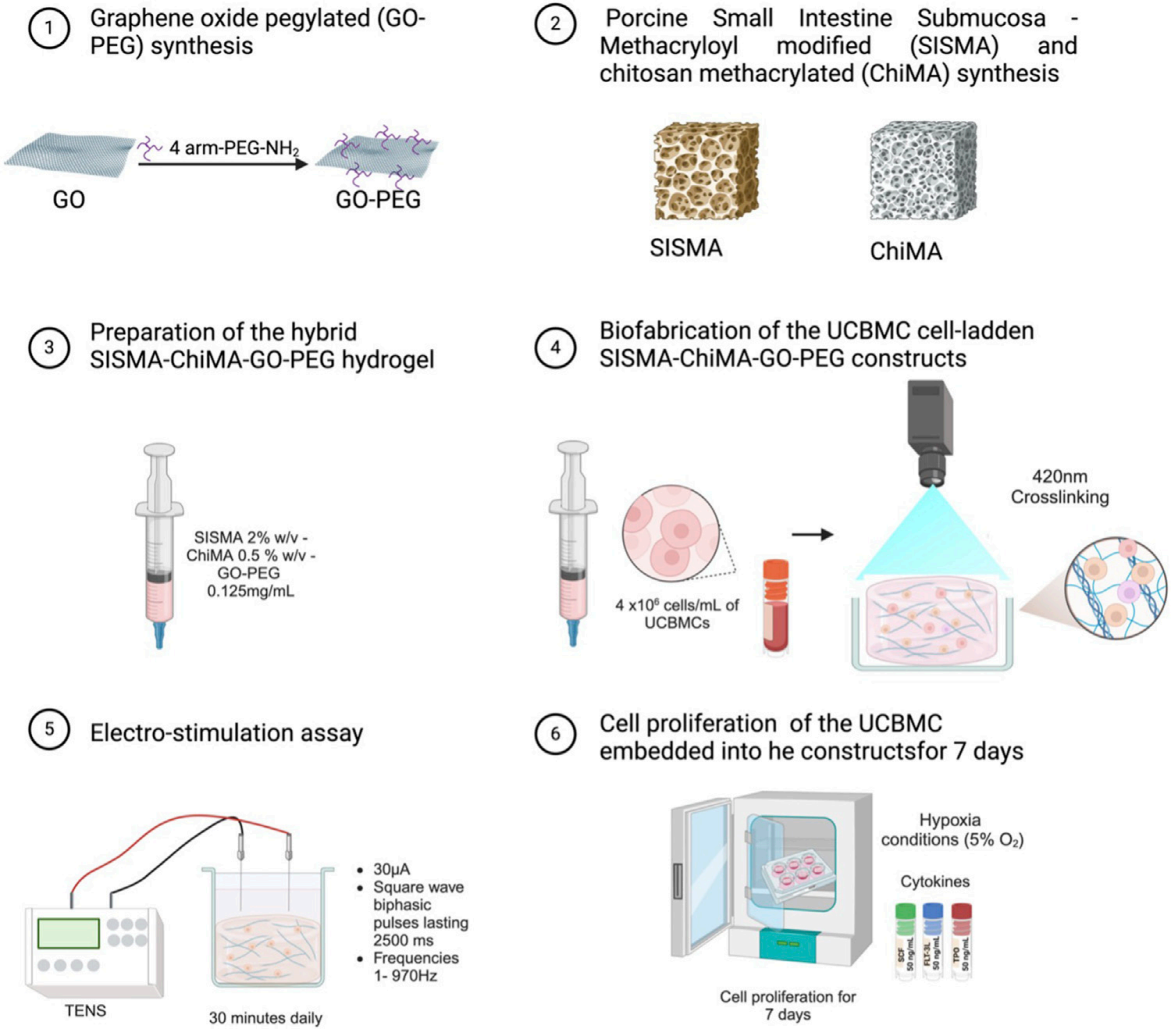
Schematic representation of the bioink preparation process, followed by the application of electrical stimulation using a TENS machine and subsequent cell maturation stages for 3D *in vitro* cultures.

An electrostimulation experiment was conducted using a transcutaneous electrical nerve stimulation machine (TENS) (Inspirstar IS02BA Microcurrent Stimulator) to deliver microcurrent stimulation via sterile acupuncture needles (Figure 1). Initially, the TENS machine and electric alligators were disinfected with 70% (v/v) ethanol solution. Separately, a custom acrylic lid for a 24-well plate, featuring four equidistant points for each well to facilitate placement of the needles, was disinfected with alkazyme and 70% (v/v) ethanol solution. This acrylic lid was designed using Autodesk Inventor Professional 2020 (Autodesk, Inc., USA, www.autodesk.com), Supplementary Figure S2. Then, the acrylic lid was placed on the 24-well plate. Two sterile acupuncture needles were placed in each well. Then, the electric alligator clips were connected to the acupuncture needles to establish the connection with the TENS machine. Subsequently, the TENS instrument was set at 30 µA of current, square wave biphasic pulses lasting 2,500 ms, and frequencies ranging from 1 Hz to 970 Hz. Subsequently, the electrostimulation was started for 15 min per day over a period of 7 days. After the electrostimulation, the needles were carefully removed, and the lid of the 24-well plate was carefully replaced. Finally, the incubation process was continued under 5% CO_2_ and 5% O_2_ (hypoxic conditions).

#### 2.7.5 Characterization of UCBMC cell viability

##### 2.7.5.1 Fluorescence staining (qualitative)

A live/dead viability staining kit was used to evaluate the viability of UCBMCs within the electrostimulated constructs. The constructs were incubated in a staining solution containing calcein-AM and propidium iodide to distinguish live (green fluorescence) and dead (red fluorescence) cells. Initially, the cells within the constructs were incubated for 45 min at 37°C under 5% CO_2_ with the solution of calcein-AM (excited by a 488 nm laser) (1:1000 in PBS 1X) and propidium iodide (excited by a 559 nm laser) (1:2000 in PBS 1X) to stain dead cells. After the incubation, the stained cells within the constructs were visualized and captured using confocal microscopy. Seventy images at 60× magnification were acquired, covering adjacent Z-planes to ensure coverage of the construct’s volume by taking sequential Z-plane images spaced by 1 µm. Finally, the proportion of live *versus* dead cells was quantified within the constructs, providing a measure of the bioink’s support for cell viability post-electrostimulation. This analysis was initially performed on day 1 and then repeated on day 7 post-maturation to monitor changes in cell viability over time.

##### 2.7.5.2 Viability assay by alamarBlue™ (quantitative)

The electrostimulated bioengineered constructs were assessed for viability assessment using the alamarBlue™ assay, a reliable indicator of cellular metabolic activity reflecting cell viability and proliferation. Initially, the alamarBlue™ reagent was dispensed into each sample well to a final concentration of 10% (v/v) and incubated for 4 h at 37°C under 5% CO_2_. After that period, the medium containing the alamarBlue™ was collected from each well. To minimize disturbance to the cells or constructs, the medium was carefully removed and replaced with fresh medium. Then, the fluorescence intensity of the collected medium was quantified using a fluorometer set to an excitation wavelength of 560 nm and an emission wavelength of 590 nm. Finally, the fold change in viability was calculated by comparing the treated construct’s fluorescence to that of the control, providing a quantitative measure of cell viability within the constructs post-electrostimulation.

#### 2.7.6 Hypoxia-induced factor (HIF) analysis by RT-PCR

##### 2.7.6.1 RNA extraction from UCBMC

RNA was extracted on day 3 post-culture from UCBMC embedded in SISMA-ChiMA-GO-PEG constructs under three different conditions: hypoxia (5% O_2_) with electrostimulation, hypoxia (5% O_2_) without electrostimulation, and normoxia (∼21% O_2_) on day 3 post culture. The extraction process involved using 1 mL of TRIzol per sample, followed by exposure to liquid nitrogen (192°C) at least 12 h before the extraction. Subsequently, each sample was thawed and homogenized using a syringe with an 18G nozzle. The homogenized samples were then incubated at room temperature for 5 min. Following the incubation, 200 μL of chloroform was added to each sample, vortexed for 15 s, and incubated at room temperature for 3 min. The samples were then centrifuged for 15 min at 12,000 G and 4°C. The aqueous phase was carefully transferred to a new, RNase-free microtube, and 500 μL of isopropanol was added. The samples were incubated for 1 h at room temperature, followed by centrifugation for 30 min at 12,000 G and 4°C. The supernatant was discarded, and the RNA pellet was resuspended in 1 mL of 75% v/v ethanol and vortexed. After centrifugation for 5 min at 7,500G and 4°C, the supernatant was discarded, and the collected RNA pellet was air-dried by leaving the microtube open to the atmosphere for approximately 30 min. The dried pellet was then resuspended in 40 μL of nuclease-free water. The purity and concentration of the extracted RNA were determined using a nanovolume spectrophotometer, and RNA integrity was assessed by electrophoresis using a 1.5% w/v agarose gel.

##### 2.7.6.2 cDNA synthesis

cDNA synthesis was evaluated using the SuperScript^®^ VILO™ cDNA Synthesis Kit. In each cDNA synthesis reaction, 4 μL of the 5X VILO™ Reaction Mix reagent and 2 μL of the 10X SuperScript™ Enzyme Mix were combined with kjd UCBMC RNA. If necessary, DEPC-treated water was added to achieve a final reaction volume of 20 μL. The components were gently mixed in a 0.2 mL Eppendorf tube and subjected to a thermal cycler with a program comprising an initial step of 10 min at 25°C, followed by 60 min at 42°C, and concluding with 5 min at 85°C.

##### 2.7.6.3 Taqman^®^ gene (HIF-1α) expression assay qPCR

The qPCR analysis was performed utilizing the Taqman^®^ Environmental Master Mix 2.0 and the Taqman^®^ Gene Expression Assay for the hypoxia-inducible factor 1α gene (HIF-1α) with an amplicon length of 76, 3 RefSeq (NM) transcripts and FAM-MGB as a reporter-associated dye. Additionally, the housekeeping gene TATA-box-binding protein (TBP) was included in the analysis, featuring an amplicon length of 79, 1 RefSeq (NM) transcript, and FAM-MGB as a reporter-associated dye. A reaction mix was prepared for each Taqman^®^ Gene Expression Assay qPCR reaction, consisting of 5 μL of the Taqman^®^ Environmental Master Mix 2.0, 0.5 μL of the Taqman^®^ Gene Expression Assay probe for the target gene (HIF-1α or TBP), and 2.5 μL of nuclease-free water. After vortexing the mixture, 8 μL of the reaction mix was added to a MicroAmp^®^ Fast Reaction Tube and combined with 2 μL of the synthesized cDNA. The same mixture was prepared as a negative control, replacing cDNA with an equivalent volume of nuclease-free water. The qPCR procedure was conducted in a real-time PCR instrument over 40 cycles, involving the following sequential steps: 2 min at 50°C (UNG incubation), 10 min at 95°C (enzyme activation), 15 s at 95°C (denaturation), and 1 min at 60°C (anneal/extend). The relative fold gene expression level (ge) was calculated following Eq. 7, where 2 corresponds to the previously calculated theoretical value of efficiency, and the double delta of the cycle threshold ( ∆∆ Ct) corresponds to the difference between the Ct values of the target and reference gene of the target samples with respect to a reference sample.

$$ge=2^{-∆∆Ct}$$ (7)

## 3 Results and discussion

### 3.1 Physicochemical analysis

#### 3.1.1 Rheological characterization

**FIGURE 2:**
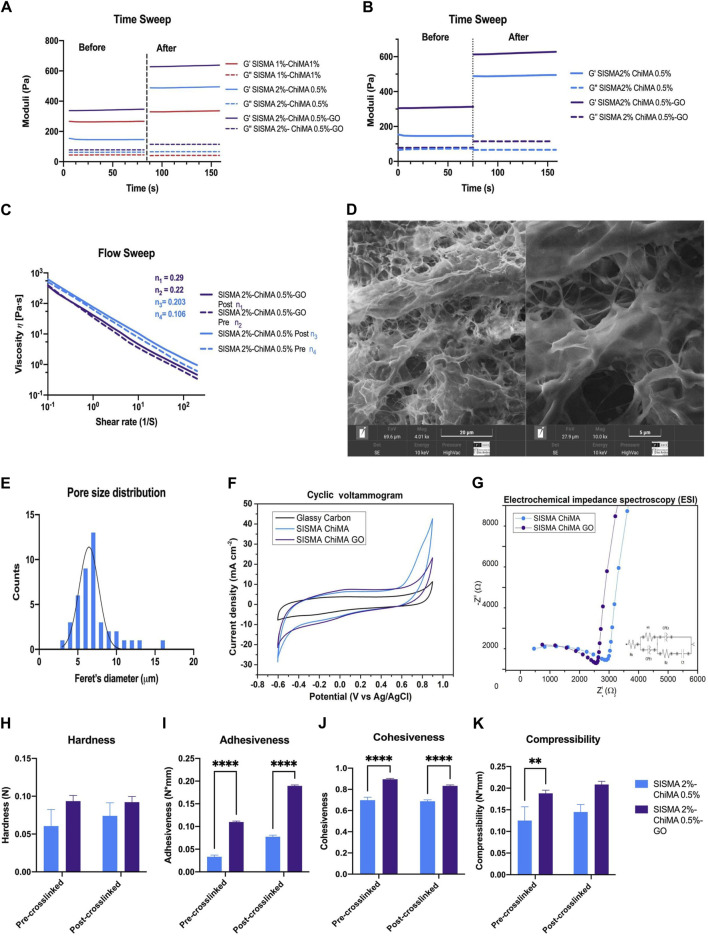
Rheological, morphological, and electrochemical characterization of SISMA 2% w/v–ChiMA 0.5% w/v and SISMA 2% w/v–ChiMA 0.5% w/v with GO-PEG 0.125 mg/mL hydrogels. **(A)** The figure shows frequency sweep analysis of SISMA at 1% w/v combined with ChiMA at 1% w/v and SISMA at 2% w/v combined with ChiMA 0.5% w/v, both before and after irradiation. The continuous lines correspond to the storage modulus (G′), and the discontinuous lines correspond to the loss modulus (G″). **(B)** Frequency sweep rheological analysis before and after irradiation. The continuous lines correspond to the storage modulus (G′), and the discontinuous lines correspond to the loss modulus (G″). **(C)** Flow sweep test results show the viscosity η vs. shear rate at 0.1–200 s^−1^ of hydrogels before and after irradiation. **(D)** SEM images of SISMA 2% w/v–ChiMA 0.5% w/v with GO-PEG 0.125 mg/mL hydrogel after photo-induced crosslinked at magnitudes of 4,010x and 10,000x. **(E)** Pore size distribution as determined by Feret’s minimum diameter from SEM images. **(F)** Cyclic voltammetry measurement of the SISMA 2% w/v–ChiMA 0.5% and w/v SISMA 2% w/v–ChiMA 0.5% w/v with GO-PEG 0.125 mg/mL hydrogels and the glassy carbon electrode. **(G)** Nyquist plots of the SISMA 2% w/v–ChiMA 0.5% and w/v SISMA 2% w/v–ChiMA 0.5% w/v with GO-PEG 0.125 mg/mL hydrogels using a Nyquist plot. Textural analysis of SISMA 2% w/v–ChiMA 0.5% w/v and SISMA 2% w/v–ChiMA 0.5% w/v with GO-PEG 0.125 mg/mL hydrogels: **(H)** hardness, **(I)** cohesiveness, **(J)** compressibility, and **(K)** adhesiveness. (Two-way ANOVA. (*) indicates *p* < 0.001, (*) indicates *p* < 0.01, and (****) indicates *p* ≤ 0.05).

After confirming the synthesis of the hydrogel components through FTIR, TGA, and Raman analyses (Supplementary Figure S3), the hybrid hydrogel was prepared by mixing SISMA, ChiMA, and GO-PEG and then physiochemically characterized. The rheological properties of the 3D matrix are crucial for its intended biomedical applications, as they impact the hydrogel’s ability to mimic the mechanical environment of biological tissues. Initially, two hydrogel formulations were analyzed: SISMA 1% w/v–ChiMA 1% w/v and SISMA 2% w/v–ChiMA 0.5% w/v (Figure 2A), aiming to select the composition that most closely mimicked the rheology behavior of bone marrow and had the best potential for 3D bioprinting and manual extrusion. Both hydrogels exhibited stable crosslinking and elastic behavior, as indicated by their storage modulus (G′) and loss modulus (G″), which remained unaffected by changes in frequency. Time sweep analyses provided insights into the mechanical integrity before and after crosslinking of the candidate formulations. The SISMA 1% w/v–ChiMA 1% w/v hydrogel showed a G′ of 260 Pa before crosslinking, which increased to around 330 Pa post crosslinking, with a consistent G″ of approximately 40 Pa. The SISMA 2% w/v–ChiMA 0.5% w/v hydrogel showed a G′ of approximately 150 Pa before crosslinking, which increased to around 500 Pa post crosslinking, with a consistent G″ of approximately 70 Pa (Figures 2A, B). After photocrosslinking, the SISMA 2% w/v–ChiMA 0.5% w/v hydrogel formulation exhibited more suitable network stability, increasing the G′ at 500 Pa compared to the 330 Pa in the SISMA 1% w/v–ChiMA 1% w/v hydrogel formulation. This result enabled us to select the second hydrogel formulation due to the mechanical stability of its polymeric network, which is mainly due to the concentration of the SISMA component (Jafarigol et al., 2020; Céspedes-Valenzuela et al., 2021). This mechanical stability was further improved by the addition of GO-PEG. In the case of the SISMA 2% w/v–ChiMA 0.5% w/v with GO-PEG 0.25 mg/mL hydrogel (Figure 2B), the G′ was approximately 300 Pa before crosslinking and increased to about 600 Pa after crosslinking, indicating enhanced mechanical stability. Interestingly, these values (∼400 Pa) were in the range observed for the bone marrow of young mice (Li et al., 2023), suggesting potential for bone tissue engineering applications. This is in contrast to other hydrogels like methacrylated hyaluronic acid (HAMA) 3% w/v, embedded with carbon nanotubes, which exhibited significantly higher G′ and G″ values (Zhang et al., 2019), and gelatin methacrylate (GelMA) 5%, known for a softer matrix with G′ values ranging from 200–300 Pa (Gilchrist et al., 2019).

Flow sweep analysis further highlighted the shear-thinning behavior of both hydrogel formulations, a desirable feature for bioinks aiming at enhanced printability (Figure 2C). Power-law analysis revealed a coefficient of n = 0.22 for the SISMA-ChiMA-GO-PEG hydrogel before crosslinking and n = 0.106 for the SISMA-ChiMA hydrogel, suggesting a pseudoplastic behavior with strong shear-thinning (close to n = 2) for the SISMA-ChiMA-GO-PEG hydrogel. This property was slightly altered post crosslinking, with n values of 0.29 for the GO-PEG integrated hydrogel and 0.203 for the SISMA-ChiMA hydrogel, affirming the pseudoplastic behavior beneficial for extrusion-based bioprinting processes. Moreover, the comparison with the rheology of intact yellow porcine bone marrow, which shows an index of n between 1.12 and 1.15, suggests that our hydrogel system offers a tunable mechanical response that can be tailored to match the properties of various tissue environments, further emphasizing its potential in regenerative medicine and tissue engineering (Jansen et al., 2015).

#### 3.1.2 Morphological characterization

In tissue engineering, the porosity and size of pores are crucial for creating an optimal environment for cell culture. These pores not only enable efficient nutrient and gas exchange, simulating physiological hypoxic conditions, but also enhance cell attachment, migration, and proliferation. The microscopic structural morphology of the freeze-dried SISMA 2% w/v–ChiMA 0.5% w/v with GO-PEG 0.25 mg/mL hydrogel was evaluated through SEM imaging. The results, shown in Figures 2D, E, reveal a homogeneous pore size distribution, with a normal distribution of Feret’s diameter ranging from 3 μm to 16 μm and peaking at 7 μm. Notably, the SEM micrographs also reveal fibrillar patterns resembling the collagen matrix present in native tissue, interspersed with discernible graphene oxide aggregates, indicating the successful integration of these components into the hydrogel matrix (Janagama and Hui, 2020; Rueda-Gensini et al., 2021).

#### 3.1.3 Electrochemical analysis

Biocompatible hydrogels with proper conductivity properties are essential for the electrical stimulation of cells. Given the similarity of the GO-PEG electroactive properties to those of its precursor, graphene oxide (GO), we explored the electrochemical behavior of GO-PEG within our SISMA-ChiMA hydrogel matrix via CV and impedance spectroscopy (EIS) using a three-electrode setup. CV is essential to the study of the redox processes of molecular species and electron transfer-initiated chemical reactions (Elgrishi et al., 2018). CV was employed to estimate the capacitance of each hydrogel. The voltammetry measurements displayed in Figure 2F demonstrate comparable capacitances for both hydrogels, each maintaining a duck-shaped curve at a scan rate of 100 mV/s. Notably, capacitive currents were observed when the potential approached 0 V. With the incorporation of GO-PEG, the SISMA-ChiMA-GO-PEG hydrogel exhibited a slightly higher capacitance value of 6.89 × 10^−3^ F/g than the 6.76 × 10^−3^ F/g of the SISMA-ChiMA hydrogel. These results confirm that embedding GO-PEG enhances the conductive properties of the SISMA-ChiMA hydrogel, as indicated by the increase in capacitance. However, it is reported that non-coated GO embedded in alginate hydrogels shows higher capacitances than protein-coated GO-containing hydrogels (as observed by Raslan et al., 2020). On the other hand, the EIS measurement in Figure 2G shows that the SISMA-ChiMA-GO-PEG hydrogel presented a shorter charge transfer resistance than the SISMA-ChiMA. The impedance circuit model indicated a charge transfer resistance (R1) for the double layer between the hydrogel and the electrolyte of 3.71 kΩ for SISMA-ChiMA and 3.47 kΩ for SISMA-ChiMA-GO-PEG, with chi-squared values of 0.13 and 0.17, respectively (Table 1) (Dutta et al., 2023). We observe that the decrease in impedance, along with the capacitance measurements, indicates an enhancement in the conductivity of the SISMA-ChiMA hydrogel after embedding GO-PEG.

**TABLE 1:** Specific capacitance and impedance parameters for the tested hydrogels.

Hydrogel	Specific capacitance C_s_ (F/g)	Rs Ω)	R1 (kΩ)	R2 (kΩ)	C1 (nF)	χ^2^
SISMA–ChiMA	5.76 × 10^−3^	400	3.71	1.02	812	0.13
SISMA–ChiMA-GO	6.89 × 10^−3^	401	3.47	1.19	838	0.17

#### 3.1.4 Texture-profile analysis

Texture-profile analysis elucidates the hydrogel’s key mechanical traits, such as hardness, adhesiveness, cohesiveness, and compressibility, which are pivotal for biomedical applications (Hurler et al., 2012). The hardness values for the SISMA 2% w/v–ChiMA 0.5% w/v and SISMA 2% w/v–ChiMA 0.5% w/v with GO-PEG 0.25 mg/mL hydrogels prior to crosslinking were approximately 0.06 N and 0.094N, respectively. Hardness measures the mechanical resistance to penetration (Hurler et al., 2012), and it is determined as the maximum peak force during the first cycle of compression. Post crosslinking, these values slightly improved to around 0.074N and 0.092 N (Figure 2H), enhancing the hydrogel’s ability to adhere to surfaces. Importantly, GO contributed to the structural stability of the hydrogels (Cépedes-Valenzuela et al., 2021; Yaprak et al., 2009). Adhesiveness, representing the work needed to overcome the attractive forces between the hydrogel surface and the probe (Hurler et al., 2012), demonstrated significantly higher values in the SISMA-ChiMA-GO-PEG hydrogel than the SISMA-ChiMA hydrogel, as depicted in Figure 2I. This increase is attributed to the addition of GO and is likely to provide stronger surface bonding, which is essential for cellular adherence (Yaprak et al., 2009). Cohesiveness, determined by the ratio of the force curve under deformation (Hurler et al., 2012), was more pronounced in the SISMA-ChiMA-GO-PEG hydrogel (Figure 2J), facilitating manipulation. While cohesiveness saw a minor decrease post crosslinking, potentially impacting the hydrogel’s ability to retain its form upon deformation, the altered compressibility underscores GO’s significant influence on the hydrogel’s textural properties, as shown in Figure 2K (Tai, Bianchini, and Jachowicz, 2014; Céspedes-Valenzuela et al., 2021). These findings collectively underscore the role of graphene oxide in influencing the mechanical properties and performance characteristics of the hydrogel, providing valuable insights for applications requiring tailored textural and mechanical attributes.

### 3.2 Proof-of-concept: Bioink preparation and electrostimulation assay

#### 3.2.1 Oxygen condition analysis

**FIGURE 3:**
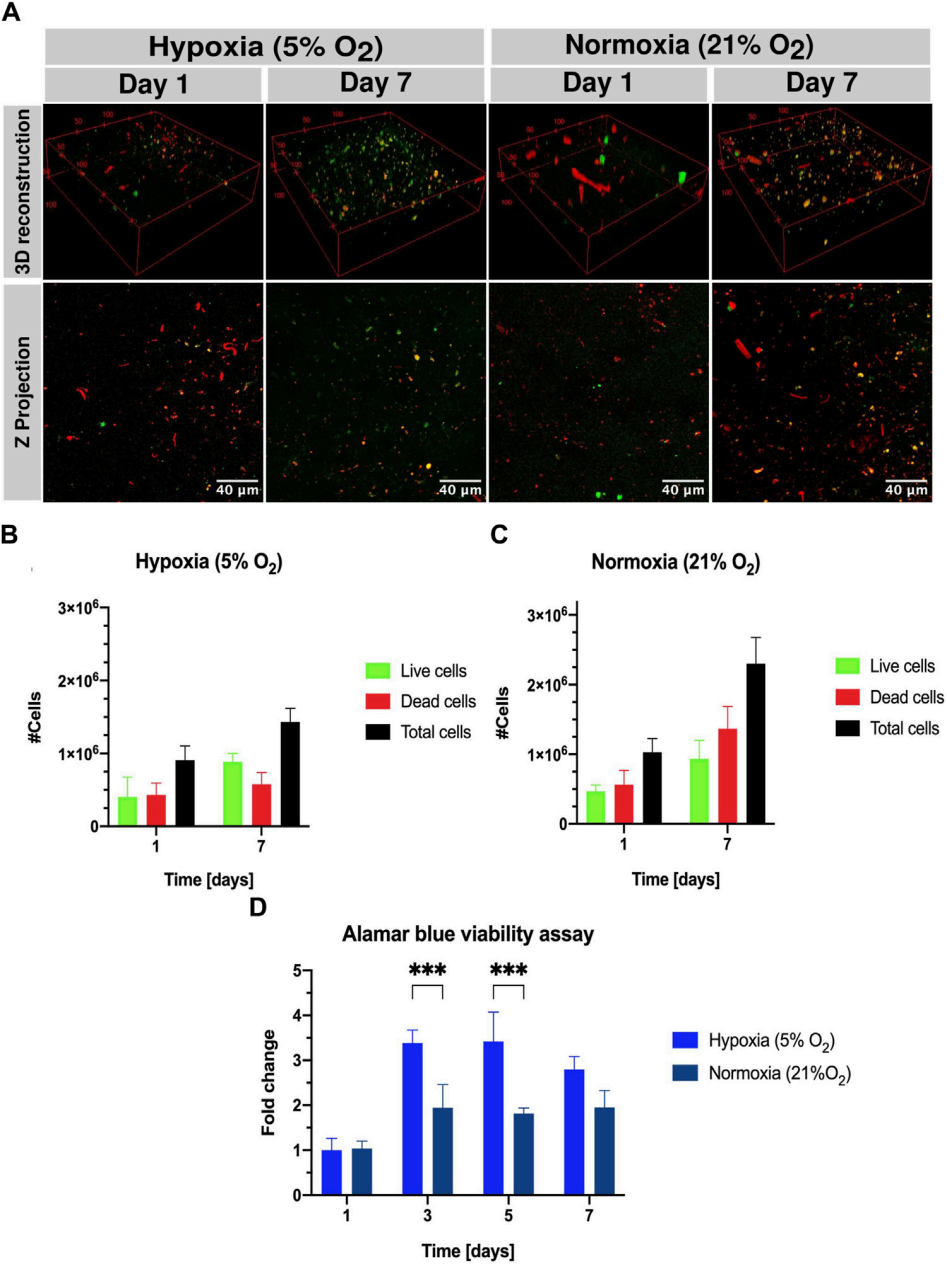
Qualitative (live/dead kit) and quantitative viability assessment (alamarBlue™ staining) of UCBMCs in SISMA-ChiMA-GO-PEG hydrogel constructs under normoxic (∼21% O_2_) and hypoxic (5% O_2_) conditions on days 1 and 7 post culture. **(A)** Confocal microscopy images capturing live cells (green fluorescence) in constructs subjected to hypoxia (5% O2) and normoxia (∼21% O_2_) on days 1 and 7 post culture. **(B)** Count of live cells, dead cells, and total cells under hypoxia (5% O2) and normoxia (∼21% O_2_). **(C)** Count of live cells, dead cells, and total cells under hypoxia (5% O2) and normoxia (∼21% O_2_). **(D)** alamarBlue™ assay results from SISMA-ChiMA-GO-PEG 3D construct culture under hypoxia (5% O2) and normoxia (∼21% O2). *p*-value ≤0.05 (*), *p*-value ≤0.01 (**).

UCBMCs were incorporated within constructs based on SISMA-ChiMA-GO-PEG at a concentration of 4 × 10^5^ cells/mL. These constructs were exposed to either hypoxic (5% O_2_) or normoxic (21% O_2_) conditions. Cell viability was assessed through qualitative (live/dead kit) and quantitative (alamarBlue™ assay) approaches, which are visualized in Figure 3. It revealed that constructs under hypoxic (5% O_2_) conditions exhibited a lower mortality of the cell population by day 7. Regarding the occurrence of live cells, hypoxic (5% O_2_) and normoxic (21% O_2_) conditions remained stable ∼9 × 10^5^ cells (Figure 3). Notably, as shown in Figure 3D, the alamarBlue™ viability assay highlighted a threefold increase observed on day 3 under hypoxic conditions, while there is a twofold increase under normoxic (21% O_2_) conditions. A noteworthy observation was the difference in cell fate between the normoxic and hypoxic conditions.

#### 3.2.2 Electrostimulation assay

In this study, we explored the impact of electrical stimulation on the proliferation of umbilical cord blood mononuclear cells (UCBMCs) within a 3D hydrogel matrix composed of SISMA, ChiMA, and GO-PEG in the presence of stem cell factor (SCF), FMS-like tyrosine kinase-3 ligand (FLT-3L), and thrombopoietin (TPO), and a hypoxic environment (5% O_2_). These early growth factors are crucial for maintaining UCBMCs in culture conditions, each playing a distinct role in supporting hematopoietic stem and progenitor cells (HSPCs): SCF enhances cell viability, TPO minimizes apoptosis and boosts megakaryocyte colony formation, and FLT-3L promotes progenitor cell proliferation (Hassan and Zander, 1996; Silnicka, 1996; Neipp et al., 1998). The hypoxic condition is employed to simulate the native bone marrow environment, fostering a quiescent state in HSPCs, maintaining low oxidative stress to prevent self-depletion or differentiation, and augmenting glycolysis to generate energy through anaerobic metabolism (Takubo et al., 2010).

**FIGURE 4:**
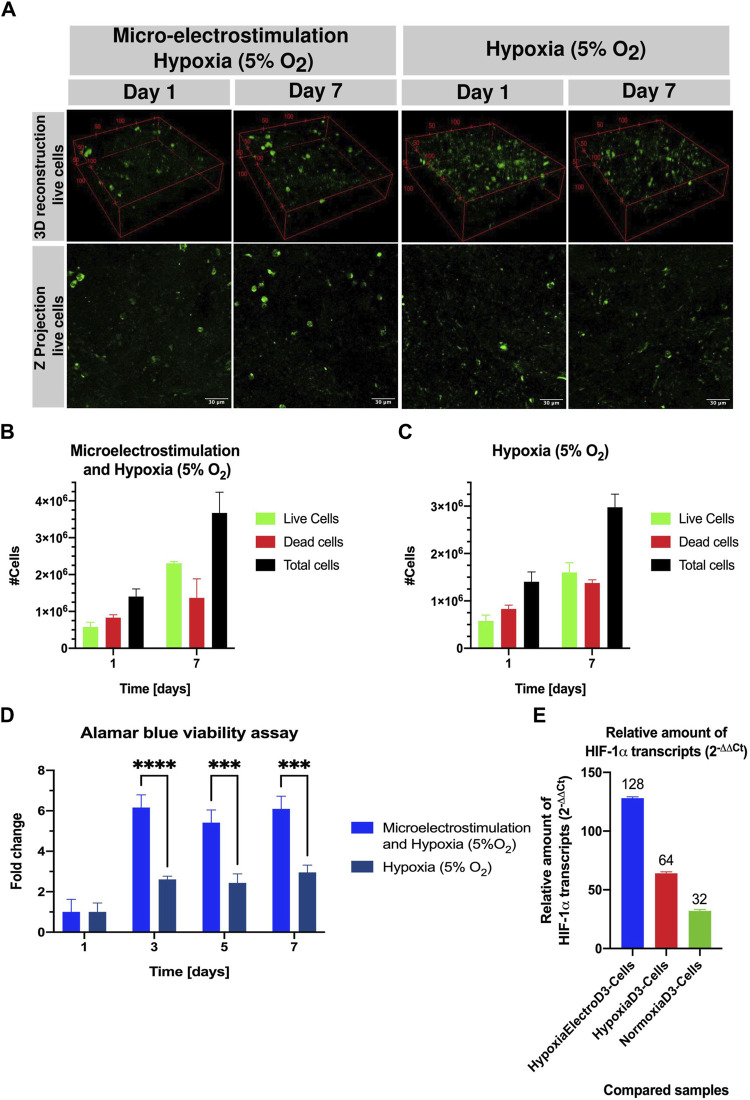
Qualitative (live/dead kit) and quantitative viability assessment (Alamar blue staining) of UCBMCs in SISMA-ChiMA-GO-PEG hydrogel constructs under micro-electrostimulation and hypoxic conditions at days 1 and 7 post culture. **(A)** Confocal microscopy images capturing live cells (green fluorescence) in constructs subjected to hypoxia (5% O_2_) with and without micro-electrostimulation on days 1 and 7 post-culture. **(B)** Count of live cells, dead cells, and total cells under micro-electrostimulation and hypoxia (5% O2). **(C)** Count of live cells, dead cells, and total cells under hypoxia (5% O_2_). **(D)** Alamar blue assay results from SISMA-ChiMA-GO-PEG 3D constructs culture under micro-electrostimulation and hypoxia (5% O_2_. **(E)** Relative amount of gene expression HIF-1⍺ transcripts in the compared samples: constructs under hypoxia (5% O_2_) and micro-electro stimulated ad day 3 vs Cells suspension, constructs under hypoxia (5% O_2_) vs Cells suspension, and constructs under normoxia (21% O_2_) vs Cells suspension. *p*-value ≤0.05 (*), *p*-value ≤0.01 (**).

Our electrostimulation experiments aimed to evaluate the potential of electrical cues to augment cell proliferation within the bioengineered constructs. Constructs embedded with UCBMCs at a concentration of 4 × 10^5^ cells/mL were subjected to micro-electrostimulation in a hypoxic setting, compared to a control group without stimulation. Cell viability assessment employing both qualitative (live/dead kit) and quantitative (alamarBlue™ assay) approaches revealed that electrostimulated constructs exhibited a significant increase in the cell population, reaching 2.3 × 10^6^ cells by day 7, compared to 1.6 × 10^6^ cells in the hypoxia-only group (Figure 4). Notably, as shown in Figure 4D, the alamarBlue™ assay highlighted a sixfold increase in viability on day three under micro-electrostimulation *versus* a threefold increase without it. Finally, a TaqMan gene expression assay qPCR was performed on the assay probes for the target genes, TBP and HIF-1α, to elucidate the impact of hypoxic (5% O_2_) and normoxic (21% O_2_) conditions, as well as micro-electrostimulation, on the gene regulation of hypoxia-inducible factor-1-alph (HIF-1α). HIF-1α is a critical transcription factor in the regulatory pathway of hypoxic culture, influencing HSPCs through the involvement of the heat shock protein GRP78 and its ligand Cripto (Suda et al., 2011). The qPCR analysis was conducted on the freshly thawed UCBMCs on day 3 of the constructs under both hypoxic (5% O_2_) and normoxic (21% O_2_) conditions and micro-electrostimulation, with day three chosen due to its peak in cell growth. The relative expression levels of HIF-1α were notably higher in samples under hypoxia (5% O_2_) than in normoxia (21% O_2_). Surprisingly, the sample of the micro-electrostimulation-simulated under hypoxic (5% O_2_) conditions exhibited the highest expression levels of HIF-1α, as shown in Figure 4E, followed by the sample under hypoxia (5% O_2_). Unexpectedly, the normoxia (21% O_2_) sample on day 3 showed a relatively high expression level of HIF-1α, potentially attributed to the 3D culture of the SISMA-ChiMA-GO hydrogel. This phenomenon may mimic a gradient of the hypoxic environment within the constructs, resembling the endosteal niche found in the bone marrow. These findings underscore the significance of HIF-1α expression: when the levels are down, HSPCs are released from the quiescent cell cycle state, leading to gradual aging and loss of the transplantation capacity (Suda et al., 2011).

These findings underscore the role of electrical stimulation in enhancing proliferative signaling pathways, notably involving the upregulation of cell nuclear antigen and the activation of ERK1/2, a key regulator of cell metabolism, motility, and proliferation (Zhou and Vijayavenkataraman, 2021). Previous research indicates the critical role of ERK1/2 in hematopoietic cell function, with its absence linked to diminished bone marrow viability. Reconstitution studies further indicate that ERK 1/2 plays redundant and kinase-dependent functions in hematopoietic progenitor cells (Chan et al., 2013). The observed increases in cell proliferation and viability highlight the promising application of electrical cues in tissue engineering. Future investigations are warranted to further explore differentiation pathways and cell lineage commitments, offering insights into the broader utility of electrostimulation in regenerative medicine. In the realm of cardiotissue engineering, electrical cues hold promise for application, with conductive hydrogels poised to play a crucial role in fostering the development of both conductive and contractile properties within constructs (Ahadian et al., 2016)**.**

## 4 Conclusion

This study has successfully developed and characterized a conductive hybrid hydrogel, incorporating methacryloyl-modified SIS decellularized extracellular matrix (SISMA), chitosan methacrylate (ChiMA), and graphene oxide-polyethylene glycol (GO-PEG), designed for the 3D *in vitro* expansion of umbilical cord blood cells (UCBMCs). Our comprehensive physicochemical characterization has revealed that this hydrogel not only maintains stable crosslinking and exhibits elastic behavior but also demonstrates shear-thinning properties. These features are vital for bioink printability and closely mimic the natural bone marrow tissue environment, potentially enhancing cell culture outcomes.

The morphological analysis of the hydrogel showed a uniform pore size distribution, which is critical for promoting effective nutrient exchange and supporting cell migration—a key aspect for tissue engineering applications. Additionally, our electrochemical analysis highlighted the hydrogel’s improved conductivity with the integration of GO-PEG, a property crucial for facilitating electrical stimulation’s beneficial effects on cell proliferation. The application of TENS within this hydrogel matrix has yielded significant enhancements in cell proliferation, particularly when the constructs were exposed to microcurrents in conjunction with early growth factors. These findings underscore the potential of electrical stimulation as a viable strategy to promote UCBC proliferation within engineered tissues.

Looking forward, our future efforts will focus on unraveling the specific molecular and cellular mechanisms by which electrical stimulation influences cell behavior within this novel hydrogel matrix. A deeper understanding of these regulatory pathways will be instrumental in optimizing the hydrogel’s design and function, paving the way for its application in regenerative medicine and tissue engineering. Through such endeavors, we aim to enhance the efficacy of 3D bioprinted constructs for a wide array of biomedical applications, further bridging the gap between *in vitro* models and clinical realities.

## Data Availability

The raw data supporting the conclusion of this article will be made available by the authors, without undue reservation.

## Supplementary material

SUPPLEMENTARY FIGURE S1 Texture profile of hydrogels before and after crosslinking. **(A)** SISMA 2% ChiMA 0.5% hydrogel. **(B)** SISMA 2% ChiMA 0.5% hydrogel with GO 0.25 mg/mL.

SUPPLEMENTARY FIGURE S2 Illustration of the custom-designed acrylic lid for a 24-well plate, created with Autodesk Inventor Professional 2020 (Autodesk, Inc., USA, www.autodesk.com). This design was specifically optimized for laser cutting to ensure an exact fit and facilitate the precise placement and securement of electrodes for electrical stimulation experiments.

SUPPLEMENTARY FIGURE S3 Characterization of graphite, GO, GO-PEG SISMA, and ChiMA. **(A)** FTIR spectra of graphite, GO, and GO-PEG. **(B)** Raman spectra of graphite, GO, and GO-PEG. **(C)** D) FTIR spectra of SISMA and ChiMA.
