# Supplementary material for: Conductive extracellular matrix derived/chitosan methacrylate/ graphene oxide-pegylated hybrid hydrogel for cell expansion
Source: Front Bioeng Biotechnol. 2024 Jun 17;12:1398052. doi: 10.3389/fbioe.2024.1398052 (PMC11215370; doi:10.3389/fbioe.2024.1398052)
Supplement: Supplementary file 1 [file DataSheet1.docx]

Supplementary Material

## Supplementary Figures


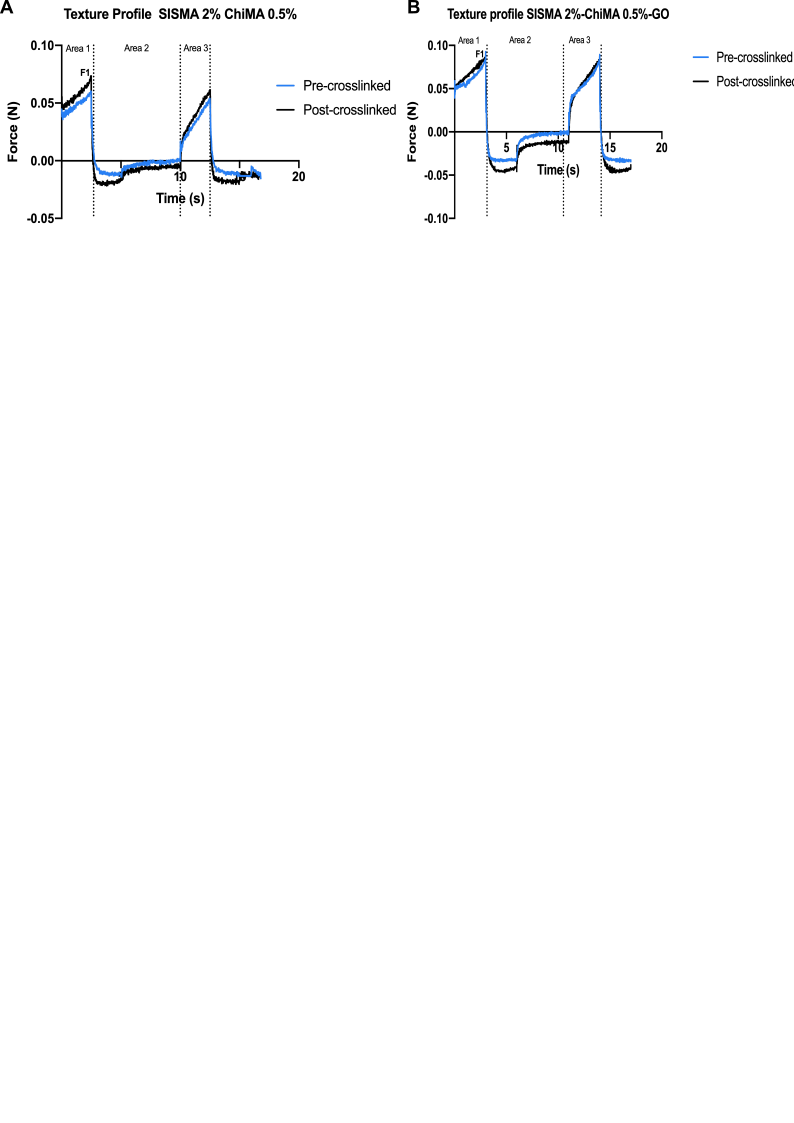


**Figure S1.** Texture profile of hydrogels pre-crosslinked and post-crosslinked. A) SISMA 2% ChiMA 0.5% hydrogel. B) SISMA 2% ChiMA 0.5% hydrogel with GO 0.25mg/mL.


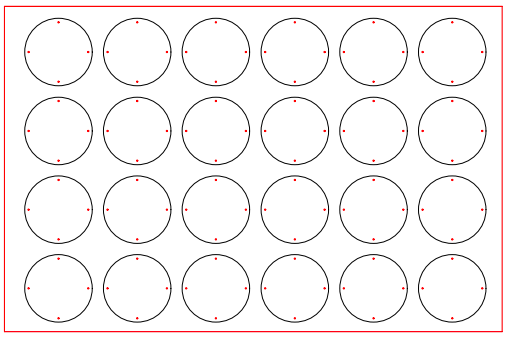


**Figure S2.** Illustration of the custom-designed acrylic lid for a 24 well-plate, created with Autodesk Inventor Professional 2020 (Autodesk, Inc., USA, [www.autodesk.com](http://www.autodesk.com)). This design was specifically optimized for laser cutting to ensure an exact fit and facilitate the precise placement and securement of electrodes for electrical stimulation experiments.


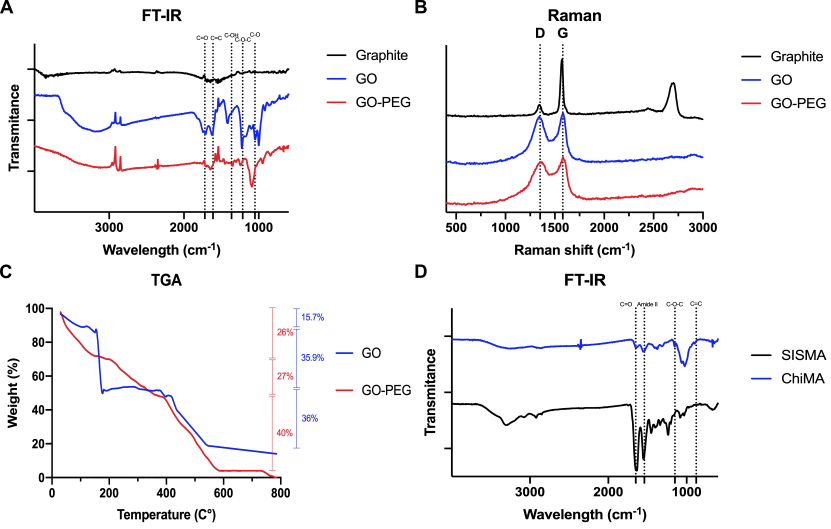


**Figure S3.** Characterization of graphite, GO, GO-PEG SISMA, and ChiMA. A) FT-IR spectra of graphite, GO, and GO-PEG. B) Raman spectra of graphite, GO and GO-PEG. C) D) FT-IR spectra of SISMA and ChiMA.
